# HATCH Score and Left Atrial Size Predict Atrial High-Rate Episodes in Patients With Cardiac Implantable Electronic Devices

**DOI:** 10.3389/fcvm.2021.746225

**Published:** 2021-10-06

**Authors:** Ju-Yi Chen, Tse-Wei Chen, Wei-Da Lu

**Affiliations:** Department of Internal Medicine, National Cheng Kung University Hospital, College of Medicine, National Cheng Kung University, Tainan, Taiwan

**Keywords:** atrial high-rate episodes, cardiac implantable electronic device, major adverse cardio/cerebrovascular events, left atrial enlargement, atrial fibrillation, HATCH score, C_2_HEST score

## Abstract

**Background:** Patients with sustained atrial high-rate episodes (AHRE) have a high risk of major adverse cardio/cerebrovascular events (MACCE). However, the prediction model and factors for the occurrence of AHRE are unknown. We aimed to identify independent factors and various risk models for predicting MACCE and AHRE.

**Methods:** We retrospectively enrolled 314 consecutive patients who had cardiac implantable electronic devices (CIEDs). The primary endpoint was MACCE after AHRE ≥3, 6 min, and 6 h. Atrial high-rate episodes was defined as >175 bpm (Medtronic^®^) lasting ≥30 s. Multivariate Cox and logistic regression analysis with time-dependent covariates were used to determine variables associated with independent risk of MACCE and occurrence of AHRE ≥3 min, respectively.

**Results:** One hundred twenty-five patients (39.8%) developed AHRE ≥3 min, 103 (32.8%) ≥6 min, and 55 (17.5%) ≥6 h. During follow-up (median 32 months), 77 MACCE occurred (incidence 9.20/100 patient years, 95% CI 5.66–18.39). The optimal AHRE cutoff value was 3 min for MACCE, with highest Youden index 1.350 (AUC, 0.716; 95% CI, 0.638–0.793; *p* < 0.001). Atrial high-rate episodes ≥3 min−6 h were independently associated with MACCE. HATCH score and left atrial diameter were independently associated with AHRE ≥3 min. The optimal cutoff for HATCH score was 3 and for left atrial diameter was 4 cm for AHRE ≥3 min.

**Conclusion:** Patients with CIEDs who develop AHRE ≥3 min have an independently increased risk of MACCE. Comprehensive assessment using HATCH score and echocardiography of patients with CIEDs is warranted.

## Introduction

All cardiac implantable electronic devices (CIEDs), including dual-chamber pacemakers; dual-chamber implantable cardioverter defibrillators; cardiac resynchronization therapy; and resynchronization-defibrillator, if an atrial lead is present, may record atrial tachyarrhythmias. Pacemaker-detected atrial high-rate episodes (AHRE), are predictors for atrial fibrillation (AF) (1) and major cardiovascular events (MACCE), including myocardial infarction, coronary revascularization, ventricular tachyarrhythmia, cardiovascular or heart failure hospitalization, and cardiovascular death (2–7). Therefore, the latest guidelines (8) recommend that AHRE be closely monitored and treated. However, the prediction of CIED-detected AHRE for MACCE has not been sufficiently assessed.

The cutoff value for AHRE duration that is associated with increased risk of MACCE remains controversial. The European Society of Cardiology guidelines state that non-valvular AF (8) with AHRE >5–6 min and >180 bpm increase the risk for ischemic stroke, but the risk for MACCE is unknown. Atrial high-rate episodes lasting ≥30 s (2) also has been shown associated with increased risk of stroke. These differences in cutoff values suggest that patients with implanted CIEDs should undergo regular assessment for detection of AHRE (8), and those with AHRE should undergo further rhythm assessment (including long-term electrocardiographic monitoring) for MACCE risk factors.

Several risk scoring systems for predicting AF, including CHA_2_DS_2_-VASc score (9), C_2_HEST score (10), and HATCH (11, 12) have been evaluated, but only C_2_HEST score has been evaluated for sustained AHRE >24 h (13). Independent predictors for AHRE in patients with dual-chamber pacemakers include sick sinus syndrome (14), increased left atrial diameter (14, 15), paced QRS duration (15), prior AF and inflammatory markers (16), and C_2_HEST score (13). However, a meta-analysis of 28 studies with 24,984 patients revealed that patients' baseline characteristics of advanced age, lower resting heart rate, diabetes, hypertension, coronary artery disease, stroke and thromboembolic events, congestive heart failure, increased left atrial diameter, and even CHADS_2_ scores were not associated with device-detected AHRE (17). These results suggest that predictors of AHRE are not well-established, and additional study is needed to identify independent predictors.

The present study investigated the optimal cutoff durations of AHRE for MACCE in patients who had CIEDs but no history of AF, and assessed independent predictive factors and validation of risk-prediction scoring systems (CHA_2_DS_2_-VASc score, HASBLED score, C_2_HEST score, and HATCH score) for AHRE in such patients.

## Methods

Consecutive patients aged 18 years or older who had CIEDs implanted (Medtronic^®^ dual chamber pacemaker, dual chamber implantable cardioverter defibrillator, cardiac resynchronization therapy-pacing, or cardiac resynchronization therapy-defibrillator) in the Cardiology Department of National Cheng Kung University Hospital from January 2015 to April 2021 were included. Every time of interrogation data of CIEDs of each enrolled patients were saved in a chart-record system in our hospital.

### Ethical Considerations

The protocol for this cohort study was reviewed and approved by the ethics committee of National Cheng Kung University Hospital and was conducted according to guidelines of the International Conference on Harmonization for Good Clinical Practice (B-ER-108-278). All included patients provided signed informed consent at the time of their implantation procedures for data to be recorded for later publication.

### Data Collection and Definitions

Patients' medical history and data of co-morbidities and echocardiographic criteria were collected from chart records for retrospective evaluation. Diabetes mellitus was defined by the presence of symptoms and casual plasma glucose concentration ≥200 mg/dl, fasting plasma glucose concentration ≥126 mg/dl, 2-h plasma glucose concentration ≥200 mg/dl from a 75-g oral glucose tolerance test, or taking medication for diabetes mellitus. Hypertension was defined as in-office systolic blood pressure values ≥140 mm Hg and/or diastolic blood pressure values ≥90 mm Hg or taking antihypertensive medication. Dyslipidemia was defined as low-density lipoprotein ≥140 mg/dl, high-density lipoprotein <40 mg/dl, triglycerides ≥150 mg/dl, or taking medication for dyslipidemia. Chronic kidney disease was defined as an estimated glomerular filtration rate (eGFR) <60 ml/min/1.73 m^2^ for at least 3 months. The primary endpoint for this study was the occurrence of MACCE after the date of CIED implantation, including ST-elevation myocardial infarction, non-ST-elevation myocardial infarction, unstable angina, systemic thromboembolism, sustained ventricular tachycardia/fibrillation, all-cause mortality, and cerebrovascular events, including stroke or transient ischemic attack (TIA) diagnosed by experienced neurologists. For each outcome, only the first event of that outcome in a subject was included. For the composite outcome, only the first event was included.

Atrial high-rate episodes were extracted from the devices via telemetry at each office visit (3–6 months). Atrial high-rate episodes electrograms were reviewed by at least one experienced electrophysiologist, who excluded lead noise or artifacts, far-field R-waves, paroxysmal supraventricular tachycardia, and visually confirmed AF that had been recorded as AHRE. Atrial sensitivity was programmed to 0.3 mV with bipolar sensing of Medtronic devices. Atrial high-rate episodes was defined as heart rate >175 bpm and at least 30 s of atrial tachyarrhythmia recorded by the devices on any day during the study. To evaluate the cutoff threshold for primary endpoints, AHRE was categorized by duration ≥3, ≥6 min, and ≥6 h. If patients had multiple AHREs, the longest AHRE duration was used for analysis. If a patient's longest AHRE duration was 7 min, the result was counted as AHRE ≥3 and ≥6 min.

### Statistical Analysis

Categorical variables are presented as percentages, and continuous variables are presented as means and standard deviations for normally distributed values or medians and interquartile interval for non-normally distributed values. The normal distribution for continuous variables was assessed with the Kolmogorov–Smirnov method. Pearson's chi-square test or Fisher's exact test was used to determine differences in baseline characteristics for categorical variables, and a two-sample student's *t*-test or Mann-Whitney U-test was used to analyze continuous variables. Survival was estimated by the Kaplan–Meier method, and differences in survival were evaluated with a log-rank test. Multivariate Cox regression analysis was used to identify variables associated with AHRE occurrence, reported as hazard ratios (HR) with 95% confidence intervals (CI). If the *p*-value in univariable analysis was <0.05, the parameter was entered into multivariable analysis. Indicators of AHRE ≥3 min, 6 min, and 6 h were determined separately as time-dependent covariates in multivariate Cox proportional hazards regression. Because CHA_2_DS_2_-VASc scores, HASBLED score, C_2_HEST score, and HATCH score overlapped many factors in univariate analysis, they were used as an independent factor in multivariate Cox regression analysis. The receiver-operating characteristic (ROC) area under the curve (AUC) of AHRE and the associated 95% CI were evaluated for association with MACCE after CIED implantation. The optimal cutoff values with the highest Youden index were chosen based on the results of ROC curve analysis and used to evaluate the associated values of AHRE in min for determining endpoints and the optimal cutoff values of left atrial size in cm and HATCH score for determining AHRE >3 min. For all comparisons, *p* < 0.05 was considered statistically significant. All data were analyzed using SPSS statistical package version 23.0 (SPSS Inc. Chicago, IL, USA).

## Results

Between January 1, 2014, and April 30, 2021, 453 consecutive patients who received Medtronic CIED implantation at National Cheng Kung University Hospital were recruited initially. Patients with previous AF (*n* = 139) were excluded, so the final analysis included data of 314 patients, of which 77 had experienced MACCE.

The median follow-up period was 32 months after implantation of CIEDs. Table 1 presents the patients' baseline demographic and clinical characteristics based on whether they had MACCE. Patients' median age was 73 years, and 61.8% of patients were men. Types of CIEDs were dual chamber pacemaker (220, 70.1%), dual chamber ICD (66, 21.0%), CRTP (23, 7.3%), and CRTD (5, 1.6%). The most common indication for CIED implantation was sick sinus syndrome (44.9%), followed by atrioventricular block (25.2%) and ventricular tachyarrhythmia (29.9%). Median atrial pacing was 25.0% and ventricular pacing 1.9%. High percentages of hypertension (80.6%), hyperlipidemia (76.8%), and diabetes (45.2%) suggest relatively high risk of primary endpoints for the entire study cohort. During follow-up, 125 (39.8%) patients developed AHRE ≥3 min, 103 (32.8%) developed AHRE ≥6 min, and 55 (17.5%) developed AHRE ≥6 h.

**Table 1:** Baseline characteristics of the overall study group and with/without primary endpoints.

**Variables**	**All patients (*n* = 314)**	**Primary endpoints** **MACCE^a^**		**Univariate *P*-value**
		**Yes (*N* = 77)**	**No (*N* = 237)**	
Age (years)	73 (62–81)	77 (68–84)	71 (60–79)	<0.001
**Gender**				0.023
Male	194 (61.8%)	56 (72.7%)	138 (58.2%)	
Female	120 (38.2%)	21 (27.3%)	99 (41.8%)	
BMI^b^ (kg/m^2^)	24.6 (22.5–26.3)	24.3 (21.6–26.2)	24.7 (22.6–26.5)	0.240
**Device type**				0.103
Dual chamber PM^c^	220 (70.1%)	62 (80.5%)	158 (66.7%)	
Dual chamber ICD^d^	66 (21.0%)	12 (15.6%)	54 (22.8%)	
CRTP^e^	23 (7.3%)	2 (2.6%)	21 (8.9%)	
CRTD^f^	5 (1.6%)	1 (1.3%)	4 (1.7%)	
**Primary indication**				0.106
Sick sinus syndrome	141 (44.9%)	41 (53.2%)	100 (42.2%)	
Atrioventricular block	79 (25.2%)	21 (27.3%)	58 (24.5%)	
Heart failure/VT^g^/VF^h^	94 (29.9%)	15 (19.5%)	79 (33.4%)	
Atrial pacing (%)	25.0 (5.8–71.4)	28.3 (6.3–82.5)	24.3 (5.3–70.7)	0.726
Ventricular pacing (%)	1.9 (0.2–98.3)	4.9 (0.2–94.5)	1.5 (0.2–98.4)	0.263
CHA_2_DS_2_-VASc score^i^	3 (2–4)	4 (3–4)	3 (1–4)	<0.001
HAS-BLED score^j^	2 (1–3)	3 (2–3)	2 (1–2)	<0.001
C_2_HEST score^k^	3 (1–3)	3 (2.5–4)	3 (1–3)	<0.001
HATCH score^l^	2 (1–3)	3 (2–4)	2 (1–2)	<0.001
Hypertension	253 (80.6%)	70 (90.9%)	183 (77.2%)	0.008
Diabetes mellitus	142 (45.2%)	53 (68.8%)	89 (37.6%)	<0.001
Hyperlipidemia	241 (76.8%)	72 (93.5%)	169 (71.3%)	<0.001
Chronic obstructive pulmonary disease	14 (4.5%)	5 (6.5%)	9 (3.8%)	0.319
Prior stroke	19 (6.1%)	7 (9.1%)	12 (5.1%)	0.198
Prior myocardial infarction	57 (18.2%)	25 (32.5%)	32 (13.5%)	<0.001
**Heart failure**				0.004
Preserved LVEF^m^	44 (14.0%)	13 (16.9%)	31 (13.1%)	
Reduced LVEF^m^	68 (21.7%)	26 (33.8%)	42 (17.7%)	
None	202 (64.3%)	38 (49.4%)	164 (69.2%)	
Chronic kidney disease	108 (34.4%)	44 (57.1%)	64 (27.0%)	<0.001
Chronic liver disease	15 (4.8%)	6 (7.8%)	9 (3.8%)	0.153
**Echo parameters**				
LVEF^m^ (%)	66 (53.8–73.0)	60.0 (40.0–72.0)	67.0 (58.0–73.0)	0.063
Mitral E/e'	11.0 (8.0–13.6)	12.0 (10.0–15.0)	10.6 (8.0–13.0)	<0.001
LA^n^ diameter (cm)	3.8 (3.2–4.1)	3.9 (3.5–4.3)	3.6 (3.1–4.1)	0.003
RV° systolic function (s', m/s)	12.0 (11.0–13.6)	12.0 (10.1–12.2)	12.0 (11.0–14.0)	0.016
**Drug prescribed at baseline**				
Antiplatelets	121 (38.5%)	49 (63.6%)	72 (30.4%)	<0.001
Anticoagulants	30 (9.6%)	5 (6.5%)	25 (10.5%)	0.293
Beta blockers	122 (38.9%)	33 (42.9%)	89 (37.6%)	0.407
Ivabradine	25 (8.0%)	8 (10.4%)	17 (7.2%)	0.365
Amiodarone	58 (18.5%)	17 (22.1%)	41 (17.3%)	0.348
Dronedarone	4 (1.3%)	2 (2.6%)	2 (0.8%)	0.253
Flecainide	1 (0.3%)	0 (0.0%)	1 (0.4%)	1.000
Propafenone	13 (4.1%)	3 (3.9%)	10 (4.2%)	1.000
Digoxin	5 (1.6%)	3 (3.9%)	2 (0.8%)	0.097
Non-DHP CCBs^p^	12 (3.8%)	2 (2.6%)	10 (4.2%)	0.737
RAAS^q^ inhibitors	141 (45.0%)	41 (53.2%)	100 (42.4%)	0.096
Diuretics	47 (15.0%)	19 (24.7%)	28 (11.8%)	0.006
Statins	121 (38.5%)	31 (40.3%)	90 (38.0%)	0.720
Metformin	50 (15.9%)	15 (19.5%)	35 (14.8%)	0.326
SGLT2^r^ inhibitors	13 (4.1%)	6 (7.8%)	7 (3.0%)	0.064
Follow-up duration (months)	32 (16–52)	26.0 (13.0–48.0)	34.0 (16.0–53.5)	0.077
AHRE^s^ duration **≥**3 min	125 (39.8%)	51 (66.2%)	74 (31.2%)	<0.001
AHRE^s^ duration **≥**6 min	103 (32.8%)	44 (57.1%)	59 (24.9%)	<0.001
AHRE^s^ duration **≥**6 h	55 (17.5%)	29 (37.7%)	26 (11.0%)	<0.001

*Data are presented as medians (interquartile interval) or n (%). Non–parametric continuous variables, as assessed using the Kolmogorov–Smirnov method, were analyzed using the Mann–Whitney U-test. Statistical significance is set at p <0.05*.

a *MACCE, major cardio/cerebrovascular events*.

b *BMI, body mass index*.

c *PM, pacemaker*.

d *ICD, implantable cardioverter defibrillator*.

e *CRTP, cardiac resynchronization therapy pacemaker*.

f *CRTD, cardiac resynchronization therapy defibrillator*.

g *VT, ventricular tachycardia*.

h *VF, ventricular fibrillation*.

i *CHA_2_DS_2_-Vasc score: Range 0–9. History of heart failure, hypertension, diabetes, vascular disease, age 65–74 years, and female sex each is calculated as 1 point; 75 years or older and prior stroke, TIA, or thromboembolism each is calculated as 2 points*.

j *HASBLED score: Range from 0 to 9. Point score is calculated as 1 point each for hypertension, abnormal kidney function, abnormal liver function, prior stroke, prior bleeding, or bleeding predisposition, labile international normalized ratio (INR), older than 65 years, medication usage predisposing to bleeding, and alcohol use*.

k *C_2_HEST score: Range from 0 to 8. C_2_: CAD/COPD (1 point each); H: hypertension (1 point); E: elderly (age ≥75 years, 2 points); S: systolic HF (2 points); and T: thyroid disease (hyperthyroidism, 1 point)*.

l *HATCH score: Range from 0 to 7. Hypertension, 1 point; age >75 years, 1 point; stroke or transient ischemic attack, 2 points; chronic obstructive pulmonary disease, 1 point; heart failure, 2 points*.

m *LVEF, left ventricular ejection fraction*.

n *LA, left atrium*.

*°RV, right ventricle*.

p *Non-DHP CCBs, non-dihydropyridine calcium channel blockers*.

q *RAAS, renin-angiotensin-aldosterone system*.

r *SGLT2, sodium glucose co-transporters 2*.

s *AHRE, atrial high-rate episodes*.

Components of primary endpoints, including MACCE, time to primary endpoints, incidence rates, and distribution of MACCE, are reported in Table 2. The total number of MACCE was 77 (incidence rate 9.20/100 patient-years, 95% CI 5.66–18.39). The endpoints were acute coronary syndrome, systemic thromboembolism events, and all-cause mortality. We also compared the incidence rates between the patients with or without AHRE in Table 2. The patients with AHRE had higher incidence rates of MACCE than those without AHRE.

**Table 2:** Types and incidences of major adverse cardio/cerebrovascular events in patients with or without AHRE.

**Types of MACCE^a^**	**Number**	**AHRE^b^(+) 209**	**Incidence rate (100 patient-years)**	**CI^c^ 95%**	**AHRE^b^(–) 105**	**Incidence rate (100 patient-years)**	**CI^c^ 95%**
TIA^d^	11	11	1.80	1.21–3.72	0	0	0
Ischemic stroke	7	6	0.98	0.66–2.03	1	0.38	0.24–0.79
Embolic event	2	2	0.33	0.22–0.68	0	0	0
ACS^e^	34	28	4.59	3.09–9.46	6	2.29	1.43–4.73
Sustained VT^f^/VF^g^	6	4	0.66	0.44–1.35	2	0.76	0.48–1.58
All-cause mortality	17	16	2.62	1.77–5.40	1	0.38	0.24–0.79
Total events	77	67	10.99	7.40–22.63	10	3.81	2.38–7.88

a *MACCE, major adverse cardio/cerebrovascular events*.

b *AHRE, atrial high rate episode*.

c *CI, confidence intervals*.

d *TIA, transient ischemic attack*.

e *ACS, acute coronary syndrome: including ST elevation myocardial infarction, non-ST elevation myocardial infarction, and unstable angina*.

f *VT, ventricular tachycardia*.

g *VF, ventricular fibrillation*.

### Univariate Analysis and Multivariate Cox Regression Analysis to Identify Associations Between AHRE Durations and MACCE

Univariate analysis revealed that age, male gender, hypertension, diabetes mellitus, hyperlipidemia, prior myocardial infarction, heart failure with reduced ejection fraction, chronic kidney disease, CHA_2_DS_2_-VASc score, HAS-BLED score, C_2_HEST score, and HATCH score were significantly associated with MACCE occurrence. Atrial high-rate episodes lasting ≥3, ≥6 min, and ≥6 h were each significantly associated with MACCE (Table 1). Multivariate Cox regression analysis using model A (not including CHA_2_DS_2_-VASc score, HASBLED score, C_2_HEST score, and HATCH score as a confounder) showed that AHRE ≥3 min (HR 3.216, 95% CI 1.745–5.927, *p* < 0.001), AHRE ≥6 min (HR 2.800, 95% CI 1.591–4.931, *p* < 0.001), and AHRE ≥6 h (HR 2.220, 95% CI 1.254–3.927, *p* = 0.006) were independently associated with MACCE, except in heart failure with reduced ejection fraction, which was also associated with MACCE (Table 3). In model B (which included CHA_2_DS_2_-VASc score, HASBLED score, C_2_HEST score, and HATCH score as a confounder), AHRE ≥3 min (HR 3.675, 95% CI 2.051–6.586, *p* < 0.001) was still independently associated with MACCE except in the presence of CHA_2_DS_2_-VASc scores, HASBLED score, C_2_HEST score, and HATCH score. We also demonstrated the independent role of AHRE in predicting ischemic thromboembolic events, including ischemic stroke and TIA, in the Supplementary Tables 1, 2.

Table 3Multivariate Cox regression analysis for major adverse cardio/cerebrovascular events.

**Table:** 

**Variables**	**Model A-1**			**Model A-2**			**Model A-3**		
	**HR**	**95%CI**	***p***	**HR**	**95%CI**	***p***	**HR**	**95%CI**	***p***
Age (y/o)	1.016	0.990–1.042	0.229	1.020	0.994–1.046	0.128	1.021	0.994–1.048	0.126
Gender (male)	0.870	0.498–1.521	0.625	0.939	0.536–1.645	0.825	0.879	0.503–1.535	0.650
Hypertension (yes)	1.386	0.000–4.240	0.965	1.943	0.000–4.236	0.964	1.337	0.000–2.233	0.963
Diabetes mellitus (yes)	1.754	0.945–3.256	0.075	1.764	0.948–3.283	0.073	1.713	0.913–3.212	0.094
Hyperlipidemia (yes)	0.713	0.163–3.119	0.654	0.465	0.103–2.098	0.319	0.622	0.141–2.744	0.531
Prior MI (yes)	0.860	0.429–1.726	0.672	0.990	0.490–2.002	0.979	0.806	0.389–1.669	0.561
CKD (yes)	1.592	0.885–2.863	0.121	1.505	0.837–2.706	0.172	1.296	0.702–2.392	0.406
Heart failure reduced ejection fraction (yes)	3.001	1.366–6.594	0.006	2.895	1.274–6.580	0.011	3.599	1.605–8.070	0.002
AHRE duration ≥3 min	3.216	1.745–5.927	<0.001						
AHRE duration ≥6 min				2.800	1.591–4.931	<0.001			
AHRE duration ≥6 h							2.220	1.254–3.927	0.006

**Table:** 

**Variables**	**Model B-1**			**Model B-2**			**Model B-3**			**Model B-4**		
	**HR**	**95%CI**	***p***	**HR**	**95%CI**	***p***	**HR**	**95%CI**	***p***	**HR**	**95%CI**	***p***
CHA_2_DS_2_-VASc score	1.584	1.292–1.940	<0.001									
HAS-BLED score				2.040	1.586–2.623	<0.001						
C_2_HEST score							1.354	1.179–1.555	<0.001			
HATCH score										1.789	1.500–2.135	<0.001
AHRE duration ≥3min	3.675	2.051–6.586	<0.001	3.323	1.851–5.964	<0.001	2.391	1.477–3.869	<0.001	2.680	1.463–4.907	0.001

### ROC-AUC Determination of AHRE Cutoff Values as Predictive Factors for Future MACCE and Survival Analysis

**Figure 1:**
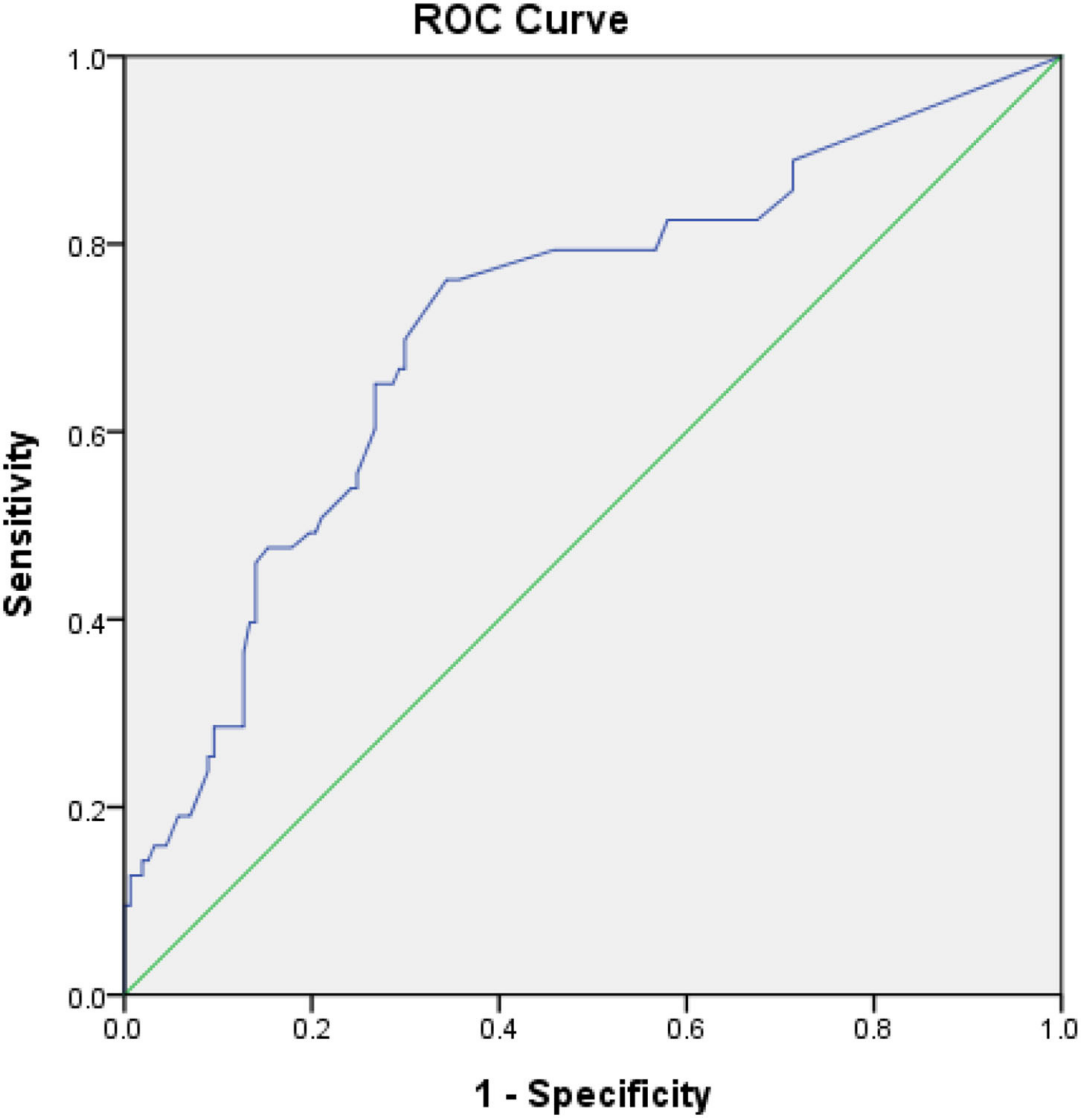
Receiver-operating characteristic curve analysis of atrial high-rate episodes (min) in patients with CIEDs with subsequent systemic embolic events. Atrial high-rate episodes (min): optimal cutoff value with the highest Youden index, 3 min; sensitivity, 76.2%; specificity, 70.0%; AUC, 0.716; 95% CI, 0.638–0.793; *p* < 0.001.

**Figure 2:**
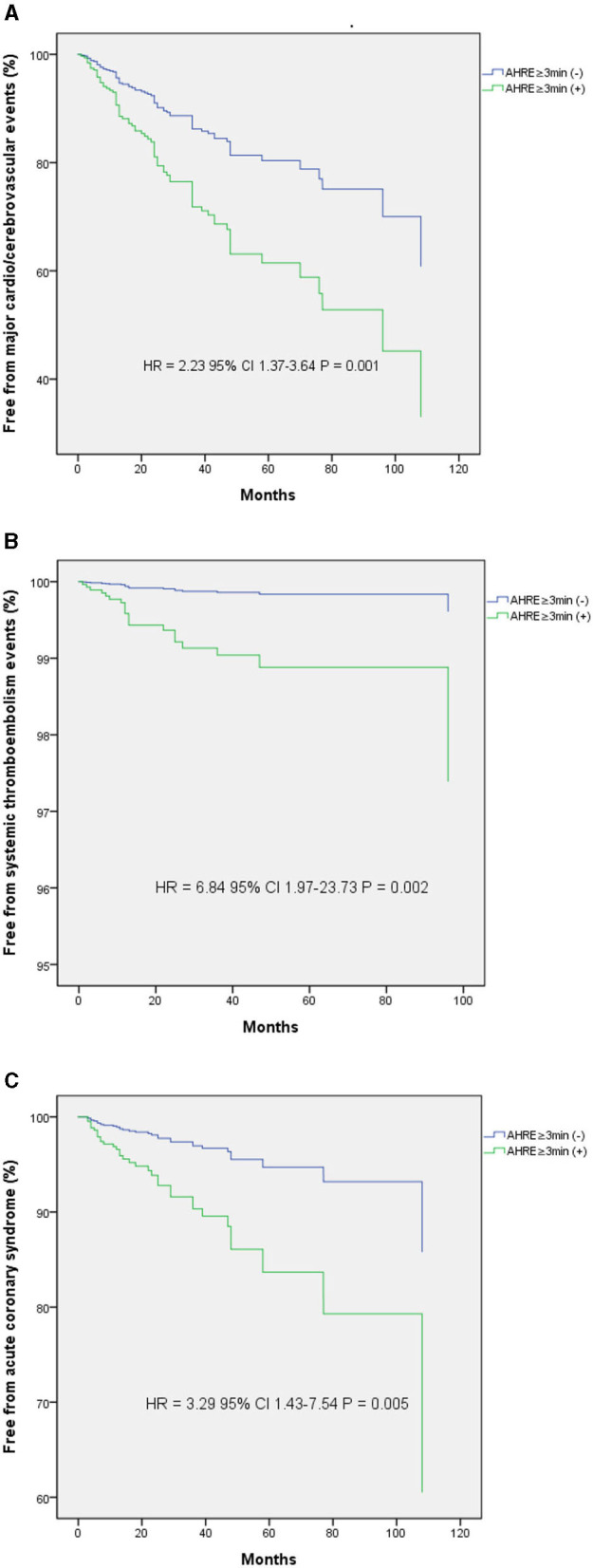
Kaplan-Meier curves depict the accumulative survival rates free from different endpoints regarding AHRE **≥**3 min or not. **(A)** For major adverse cardio/cerebrovascular events, a significant higher hazard is seen among the AHRE **≥**3 min (+) compared with the AHRE **≥**3 min (–) (HR 2.23, log-rank *p* = 0.001). **(B)** For systemic thromboembolism events, a significant higher hazard is seen among the AHRE **≥**3 min (+) compared with the AHRE **≥**3 min (–) (HR 6.84, log-rank *p* = 0.002). **(C)** For acute coronary syndrome, a significant higher hazard is seen among the AHRE **≥**3 min (+) compared with the AHRE **≥**3 min (–) (HR 3.29, log-rank *p* = 0.005).

The optimal AHRE cutoff value predictive of MACCE was determined to be 3 min, with the highest Youden index 1.350 (sensitivity, 76.2%; specificity, 70.0%; AUC, 0.716; 95% CI, 0.638–0.793; *p* < 0.001) (Figure 1). The survival analysis revealed a significant correlation between an AHRE ≥3 min detection and a shorter event-free survival time. Multivariate Cox proportional hazards analysis further revealed that AHRE ≥3 min were associated with increased risk of MACCE (HR = 2.23, 95% CI 1.37–3.64, *p* = 0.001), systemic thromboembolism events (HR = 6.84, 95% CI 1.97–23.73, *p* = 0.002), and acute coronary syndrome (HR = 3.28% CI 1.43–7.54, *p* = 0.005) (Figure 2).

### Univariate Analysis and Multivariate Logistic Regression Analysis to Identify Associations Between Risk Models and AHRE ≥3 Min

After a median follow-up of 32 months, 125 (39.8%) patients had AHRE ≥3 min. In univariate analysis, sick sinus syndrome, hypertension, hyperlipidemia, chronic kidney disease, accumulated ventricular pacing loads, left atrial diameter, mitral E/E', CHA_2_DS_2_-VASc scores, HASBLED score, C_2_HEST score, and HATCH score were significantly different between patients with or without AHRE ≥3 min (Table 4).

**Table 4:** Baseline characteristics of the overall study group and with/without AHRE ≥3 min.

**Variables**	**All patients (*n* = 314)**	**AHRE** **≥3 min**		**Univariate *P*-value**
		**Yes (*N* = 125)**	**No (*N* = 189)**	
Age (years)	72.5 (62–81)	76 (64–83)	70 (59–79)	0.006
**Gender**				0.108
Male	194 (61.8%)	84 (67.2%)	110 (58.2%)	
Female	120 (38.2%)	41 (32.8%)	79 (41.8%)	
BMI^a^ (kg/m^2^)	24.6 (22.5–26.3)	24.5 (22.3–26.0)	24.8 (22.6–26.8)	0.080
**Device type**				0.001
Dual chamber PM^b^	220 (70.1%)	102 (81.6%)	118 (62.4%)	
Dual chamber ICD^c^	66 (21.0%)	12 (9.6%)	54 (28.6%)	
CRTP^d^	23 (7.3%)	9 (7.2%)	14 (7.4%)	
CRTD^e^	5 (1.6%)	2 (1.6%)	3 (1.6%)	
**Primary indication**				0.001
Sick sinus syndrome	141 (44.9%)	62 (49.6%)	79 (41.8%)	
Atrioventricular block	79 (25.2%)	40 (32.0%)	39 (20.6%)	
Heart failure/VT^f^/VF^g^	94 (29.9%)	23 (18.4%)	71 (37.6%)	
Atrial pacing (%)	25.0 (5.8–71.4)	29.2 (4.8–70.5)	23.1 (6.2–77.6)	0.937
Ventricular pacing (%)	1.9 (0.2–98.3)	16.5 (0.5–98.9)	0.3 (0.2–86.4)	<0.001
CHA_2_DS_2_-VASc score^h^	3 (2–4)	3 (2–4)	3 (2–4)	0.027
C_2_HEST score^i^	3 (1–3)	3 (1–3)	3 (1–3)	0.016
HATCH score^j^	2 (1–3)	2 (1–3)	2 (1–2)	<0.001
Hypertension	253 (80.6%)	113 (90.4%)	140 (74.1%)	<0.001
Diabetes mellitus	142 (45.2%)	60 (48.0%)	82 (43.4%)	0.421
Hyperlipidemia	241 (76.8%)	108 (86.4%)	133 (70.4%)	0.001
Chronic obstructive pulmonary disease	14 (4.5%)	7 (5.6%)	7 (3.7%)	0.425
Prior stroke	19 (6.1%)	9 (7.2%)	10 (5.3%)	0.487
Prior myocardial infarction	57 (18.2%)	28 (22.4%)	29 (15.3%)	0.112
**Heart failure**				0.448
Preserved LVEF^k^	44 (14.0%)	21 (16.8%)	23 (12.2%)	
Reduced LVEF^k^	68 (21.7%)	28 (22.4%)	40 (21.2%)	
None	202 (64.38)	76 (60.8%)	126 (66.7%)	
Chronic kidney disease	108 (34.4%)	53 (42.4%)	55 (29.1%)	0.015
Chronic liver disease	15 (4.8%)	7 (5.6%)	8 (4.2%)	0.578
**Echo parameters**				
LVEF^k^ (%)	66.0 (53.8–73.0)	66.0 (53.5–73.0)	66.0 (52.5–73.0)	0.716
Mitral E/e'	11.0 (8.0–13.6)	11.1 (9.0–14.5)	10.7 (7.7–13.0)	0.012
LA^l^ diameter (cm)	3.8 (3.2–4.1)	3.9 (3.4–4.3)	3.6 (3.1–4.1)	0.003
RV^m^ systolic function (s', m/s)	12.0 (11.0–13.6)	12.0 (11.0–14.0)	12.0 (11.0–13.5)	0.995
**Drug prescribed at baseline**				
Antiplatelets	121 (38.5%)	52 (41.6%)	69 (36.5%)	0.364
Anticoagulants	30 (9.6%)	20 (16.0%)	10 (5.3%)	0.002
Beta blockers	122 (38.9%)	46 (36.8%)	76 (40.2%)	0.544
Ivabradine	25 (8.0%)	9 (7.2%)	16 (8.5%)	0.685
Amiodarone	58 (16.8%)	21 (16.8%)	37 (19.6%)	0.535
Dronedarone	4 (1.3%)	3 (2.4%)	1 (0.5%)	0.305
Flecainide	1 (0.3%)	0 (0.0%)	1 (0.5%)	1.000
Propafenone	13 (4.1%)	9 (7.2%)	4 (2.1%)	0.040
Digoxin	5 (1.6%)	3 (2.4%)	2 (1.1%)	0.390
Non-DHP CCBs^n^	12 (3.8%)	6 (4.8%)	6 (3.2%)	0.462
RAAS° inhibitors	141 (45.0%)	55 (44.0%)	86 (45.7%)	0.761
Diuretics	47 (15.0%)	21 (16.8%)	26 (13.8%)	0.459
Statins	121 (38.5%)	41 (32.8%)	80 (42.3%)	0.089
Metformin	50 (15.9%)	14 (11.2%)	36 (19.0%)	0.063
SGLT2^p^ inhibitors	13 (4.1%)	4 (3.2%)	9 (4.8%)	0.575

*Data are presented as medians (interquartile interval) or n (%). Non-parametric continuous variables, as assessed using the Kolmogorov–Smirnov method, were analyzed using the Mann–Whitney U test. Statistical significance is set at p <0.05*.

a *BMI, body mass index*.

b *PM, pacemaker*.

c *ICD, implantable cardioverter defibrillator*.

d *CRTP, cardiac resynchronization therapy pacemaker*.

e *CRTD, cardiac resynchronization therapy defibrillator*.

f *VT, ventricular tachycardia*.

g *VF, ventricular fibrillation*.

h *CHA_2_DS_2_-Vasc score: Range from 0 to 9. History of heart failure, hypertension, diabetes, vascular disease, age 65–74 years, and female sex each is calculated as 1 point; 75 years or older and prior stroke, TIA, or thromboembolism each is calculated as 2 points*.

i *C_2_HEST score: Range from 0 to 8. C_2_: CAD/COPD (1 point each); H: hypertension (1 point); E: elderly (age ≥75 years, 2 points); S: systolic HF (2 points); and T: thyroid disease (hyperthyroidism, 1 point)*.

j *HATCH score: Range from 0 to 7. Hypertension, 1 point; age >75 years, 1 point; stroke or transient ischemic attack, 2 points; chronic obstructive pulmonary disease, 1 point; heart failure, 2 points*.

k *LVEF, left ventricular ejection fraction*.

l *LA, left atrium*.

m *RV, right ventricle*.

n *Non-DHP CCBs, non-dihydropyridine calcium channel blockers.*

*°RAAS, renin-angiotensin-aldosterone system.*

p *SGLT2, sodium glucose co-transporters 2*.

Multivariate logistic regression analysis revealed that among values from the significant variables from the univariate analysis, only HATCH score (HR 1.546, 95% CI 1.211–1.973, *p* < 0.001) and left atrial diameter (HR 1.559, 95% CI 1.038–2.341, *p* = 0.033) were independently associated with AHRE ≥3 min (Table 5).

**Table 5:** Multivariate logistic regression analysis for independent factors of subsequent atrial high rate episodes ≥3 min.

	**Model 1**			**Model 2**			**Model 3**			**Model 4**		
	**HR**	**95%CI**	***p***	**HR**	**95%CI**	***p***	**HR**	**95%CI**	***p***	**HR**	**95%CI**	***p***
Age (y/o)	0.999	0.977–1.022	0.944									
Indications (SSS)^a^	1.160	0.610–2.209	0.650	1.161	0.606–2.224	0.653	1.224	0.638–2.350	0.543	1.354	0.698–2.624	0.370
Ventricular pacing (%)	1.002	0.996–1.009	0.493	1.002	0.996–1.009	0.495	1.002	0.996–1.009	0.504	1.002	0.995–1.009	0.642
Hypertension (yes)	1.611	0.646–4.017	0.306									
Hyperlipidemia (yes)	1.065	0.467–2.431	0.881	1.208	0.523–2.793	0.658	1.164	0.538–2.516	0.700	0.960	0.435–2.119	0.919
Chronic kidney disease (yes)	1.335	0.780–2.285	0.292	1.334	0.766–2.324	0.308	1.213	0.693–2.124	0.499	1.028	0.588–1.797	0.924
Mitral E/E'	1.017	0.970–1.067	0.486	1.019	0.971–1.070	0.450	1.016	0.968–1.066	0.524	1.002	0.953–1.053	0.935
Left atrial diameter (cm)	1.665	1.111–2.497	0.014	1.651	1.101–2.475	0.015	1.619	1.082–2.422	0.019	1.559	1.038–2.341	0.033
CHA_2_DS_2_-VASc score^b^				1.028	0.834–1.268	0.793						
C_2_HEST score^c^							1.123	0.927–1.360	0.236			
HATCH score^d^										1.546	1.211–1.973	<0.001

a *SSS: Sick sinus syndrome*.

b *CHA_2_DS_2_-Vasc score: Range from 0 to 9. History of heart failure, hypertension, diabetes, vascular disease, age 65–74 years, and female sex each is calculated as 1 point; 75 years or older and prior stroke, TIA, or thromboembolism each is calculated as 2 points*.

c *C_2_HEST score: Range from 0 to 8. C_2_: CAD/COPD (1 point each); H: hypertension (1 point); E: elderly (age ≥75 years, 2 points); S: systolic HF (2 points); and T: thyroid disease (hyperthyroidism, 1 point)*.

d *HATCH score: Range from 0 to 7. Hypertension, 1 point; age >75 years, 1 point; stroke or transient ischemic attack, 2 points; chronic obstructive pulmonary disease, 1 point; heart failure, 2 points*.

### ROC-AUC Determination of HATCH Score and Left Atrial Diameter Cutoff Values Associated With AHRE ≥3 Min

**Figure 3:**
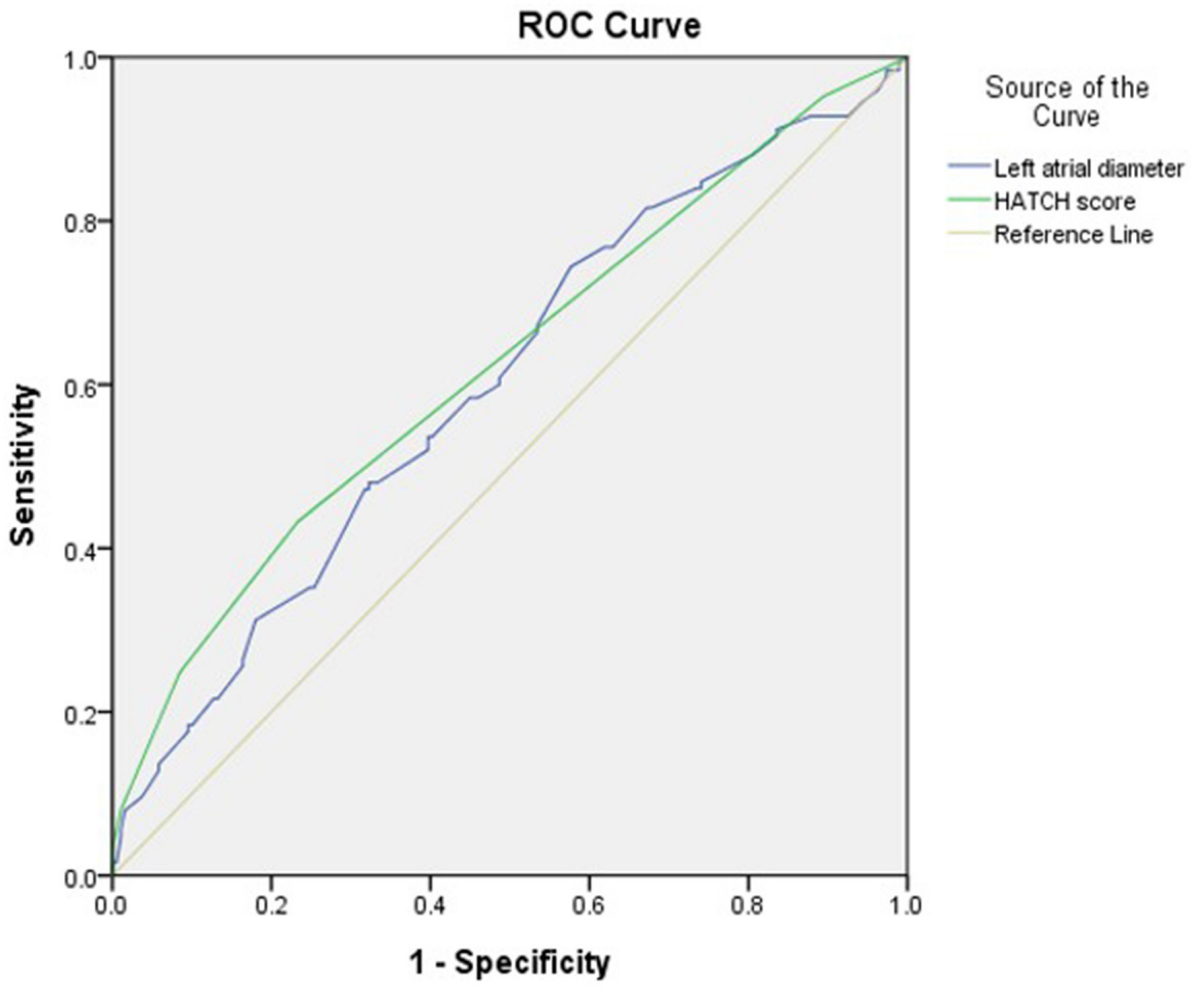
Receiver-operating characteristic curve analysis of factors in patients with CIEDs with subsequent AHRE **≥**3 min. Left atrial diameter (cm): the highest Youden index: 4.0 cm, AUC, 0.600; 95% CI, 0.536–0.664; *p* = 0.003. HATCH score: the highest Youden index: 3, AUC, 0.624; 95% CI, 0.560–0.688; *p* < 0.001.

**Figure 4:**
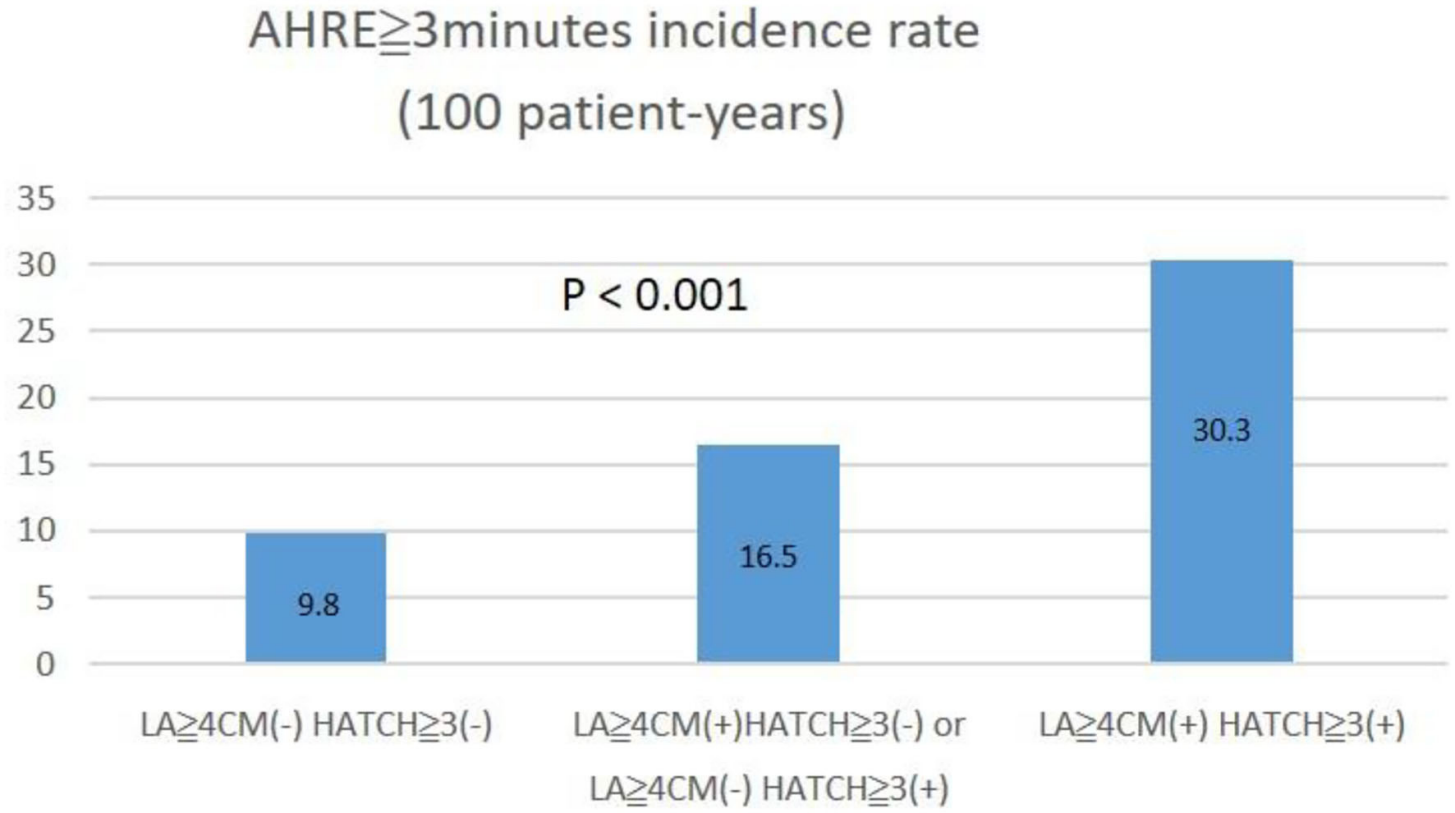
The incidence percentage of AHRE **≥**3 min significantly increased with left atrial size **≥**4 cm and HATCH score **≥**3.

The optimal HATCH score cutoff value for AHRE ≥3 min was 3, with the highest Youden index (sensitivity, 43.2%; specificity, 76.7%; AUC, 0.624; 95% CI, 0.560–0.688; *p* < 0.001) (Figure 3). The optimal left atrial diameter cutoff value for subsequent AHRE ≥3 min was 4 cm, with the highest Youden index (sensitivity, 47.2%; specificity, 68.3%; AUC, 0.600; 95% CI, 0.536–0.664; *p* = 0.003) (Figure 3). We also found that patients with both left diameter ≥4 cm and HATCH score ≥3 had a higher risk for AHRE ≥3 min than did patients with either left atrial diameter <4 cm or HATCH score <3 (Figure 4).

## Discussion

The main finding of this study is that AHRE lasting ≥3, ≥6 min, or ≥6 h is significantly and independently associated with MACCE in a Taiwanese population having CIEDs and no history of AF. The optimal cutoff value of AHRE for subsequent MACCE was 3 min. Increased left atrial diameter and HATCH score were independently associated with AHRE duration ≥3 min. These results suggest that early detection of AHRE ≥3 min and measurement of left diameter and calculation of the HATCH score in patients with CIEDs is warranted to prompt early, aggressive therapy to prevent MACCE.

This study was conducted because the optimal cutoff for AHRE duration to predict MACCE in patients with CIEDs had not been well-studied, and predictive factors were not established. Sometimes, a relative short-duration of atrial tachy-arrhythmias, such as <30 s, may be misclassified as AHRE due to artifacts and false detection of far-field R-waves by the atrial lead. European Society of Cardiology guidelines (8) recommend that AF can only be diagnosed by 12-lead electrocardiography or more than 30 s in an electrocardiographic strip. The updated guidelines (8) also recommend that if AHRE ≥6 min with high CHA_2_DS_2_-VASc score or AHRE ≥24 h occur, more aggressive monitoring of clinical AF is warranted.

Although most studies have focused on systemic embolic or neurological events occurring after AHRE, more recent studies have found that MACCE, including ventricular tachyarrhythmias (6, 7), heart failure (6), myocardial infarction (6), and cardiovascular death (6), also were associated with AHRE ≥5 min, and the association was even stronger for AHRE ≥24 h. Also, the use of different settings for AHRE detection is an important factor that can affect results between these studies. Pastori et al. (6) used 175 beats/min, and Vergara et al. (7) used 200 beats/min as threshold rate. We used 175 beats/min (Medtronic), and at least 30 s of atrial tachyarrhythmia recorded by the CIEDs on any day during the study period. Atrial high-rate episodes ≥3, ≥6 min, and ≥6 h were all significant risk factors for future MACCE. Only the present study has demonstrated that AHRE is an independent risk factor for MACCE, and the optimal cutoff value for predicting MACCE is 3 min.

Several pathophysiological mechanisms of AHRE in MACCE have been proposed (18): (1) AHRE as a precursor of AF, leading to coronary or systemic thromboembolism from the left atrium or left atrial appendage, resulting in acute coronary syndrome or neurologic events; (2) AHRE associated with multiple atherosclerotic risks and associated inflammatory process, yielding a pro-thrombotic state; and (3) AHRE resulting in a supply-demand mismatch between the coronary system and heart function. Hence, the relationship of AHRE duration and MACCE is an important area of research. Large-scale studies are needed to explore AHRE duration cutoffs, with the goal of establishing a standard cutoff for further evaluation of MACCE in patients with AHRE.

Awareness of risk factors that contribute to the occurrence of AHRE ≥3 min is important for early prevention in patients with CIEDs. Previous studies (13–17) identified several predictors for AHRE; a consistent predictive factor was increased left atrial diameter (14, 15). The Korean study (14) demonstrated that left atrial diameter >41 mm was associated with AHRE ≥6 min, and the Indian study (15) reported that increased left atrial diameter contributed to prolonged AHRE. In the present study, increased left atrial diameter was consistently and significantly associated with AHRE ≥3 min, a finding compatible with results of the two studies above. Our results suggest that evaluation of patients' echocardiographic features before implantation of CIEDs should include measurement of left atrial size, which may provide early prediction of AHRE ≥3 min—a strong predictor for MACCE.

Risk scoring systems, such as CHA_2_DS_2_-VASc score (9), C_2_HEST score (10), and HATCH score (11, 12), have been evaluated for predicting AF, but only the C_2_HEST score was evaluated for sustained AHRE >24 h (13). We found that the HATCH score independently predicted sustained AHRE ≥3 min (HR 1.546, *p* < 0.001), but the CHA_2_DS_2_-VASc score and the C_2_HEST score did not. We found also that the percentage of AHRE ≥3 min increased with increasing HATCH score. Recently, Li et al. (19), in China, modified the mC_2_HEST score (adding age ≥65 years as one point), which increased the predictive accuracy and discriminative capability for incident AF. We also evaluated the performance of the mC_2_HEST score but found it not as suitable as the HATCH score (data not shown).

## Limitations

Our study has limitations. First, this was a single-center, retrospective, observational study with a relatively small number of patients with CIEDs in a hospital setting, and all patients were Taiwanese. Thus, causality cannot be inferred between AHRE and MACCE, and the presence of confounding factors cannot be denied. Also, the results may not be generalizable to other populations. Thus, prospective multicenter studies with larger samples are required to confirm the results of this study. Second, this study did not investigate the nature of heart rhythms at the time of onset of MACCE. Third, in this retrospective analysis of patient data, we could not confirm that patients started anticoagulants due to CIED-detected AHRE, although these patients were not excluded because no significant differences were found between anticoagulants use and the presence (5, 6.5%) or absence (25, 10.5%) of MACCE (*p* = 0.293), as shown in Table 1.

## Conclusions

Major cardiovascular events are not uncommon in patients after implantation of CIEDs. Episodes of AHRE lasting ≥3 min to ≥6 h are independent risk factors for MACCE in this population during mid-term follow-up. When AHRE ≥3 min is detected in patients with CIEDs, long-term monitoring to detect clinical AF and comprehensive assessment of MACCE risk with HATCH score and echocardiography (to determine left atrial size) for risk stratification are indicated. Early detection of AHRE ≥3 min and measurement of left atrial diameter and calculation of HATCH score in patients with CIEDs may be warranted to prompt early, aggressive therapy and prevent MACCE.

## Data Availability Statement

The raw data supporting the conclusions of this article will be made available by the authors, without undue reservation.

## Ethics Statement

The studies involving human participants were reviewed and approved by Institutional Review Board of National Cheng Kung University Hospital (B-ER-108-278).

## Author Contributions

J-YC: conception and design, data analysis and interpretation, statistical analysis, drafting and finalizing the article, and critical revision of the article for important intellectual content. T-WC and W-DL: data acquisition. All authors contributed to the article and approved the submitted version.

## Funding

The authors thank the Ministry of Science and Technology of the Republic of China, Taiwan, for financially supporting this research under contract MOST 109-2218-E-006-024 and MOST 110-2218-E-006-017.

## Conflict of Interest

The authors declare that the research was conducted in the absence of any commercial or financial relationships that could be construed as a potential conflict of interest.

## Publisher's Note

All claims expressed in this article are solely those of the authors and do not necessarily represent those of their affiliated organizations, or those of the publisher, the editors and the reviewers. Any product that may be evaluated in this article, or claim that may be made by its manufacturer, is not guaranteed or endorsed by the publisher.

## Abbreviations

AF: atrial fibrillation
AHRE: atrial high-rate episodes
CIEDs: cardiac implantable electronic devices
MACCE: major adverse cardio/cerebrovascular events
TIA: transient ischemic attacks.
