# Supplementary material for: HATCH Score and Left Atrial Size Predict Atrial High-Rate Episodes in Patients With Cardiac Implantable Electronic Devices
Source: Front Cardiovasc Med. 2021 Oct 6;8:746225. doi: 10.3389/fcvm.2021.746225 (PMC8528173; doi:10.3389/fcvm.2021.746225)
Supplement: Supplementary file 1 [file Table_1.docx]

| **Supplemental Table 1. Baseline Characteristics of the Overall Study Group and with/without ischemic cerebrovascular events** | | | | |
| --- | --- | --- | --- | --- |
| **Variables** | **All Patients**  **(n=314)** | **Ischemic cerebrovascular events** | | **Univariate**  **P valve** |
|  |  |  |  |  |
|  |  | **Yes**  **(N=18)** | **No**  **(N=296)** |  |
| Age (years) | 73 (62-81) | 75 (68-83) | 72 (61-81) | 0.153 |
| Gender | | | | 0.212 |
| Male | 194(61.8%) | 14(77.8%) | 180(60.8%) |  |
| Female | 120(38.2%) | 4(22.2%) | 116(39.2%) |  |
| BMI^b^ (kg/m^2^) | 24.6(22.5-26.3) | 23.9(22.6-25.9) | 24.6(22.4-26.3) | 0.616 |
| Device type |  |  |  | 0.318 |
| Dual chamber PM^c^ | 220(70.1%) | 16(88.9%) | 204(68.9%) |  |
| Dual chamber ICD^d^ | 66(21.0%) | 1(5.6%) | 65(22.0%) |  |
| CRTP^e^ | 23(7.3%) | 1(5.6%) | 22(7.4%) |  |
| CRTD^f^ | 5(1.6%) | 0(0.0%) | 5(1.7%) |  |
| Primary Indication |  |  |  | 0.178 |
| Sinus node dysfunction | 141(44.9%) | 13(72.2%) | 128(43.2%) |  |
| Atrioventricular block | 79(25.2%) | 3(16.7%) | 76(25.7%) |  |
| Heart failure/VT^g^/VF^h^ | 94(29.9%) | 2(11.2%) | 92(31.1%) |  |
| Atrial pacing (%) | 25.0 (5.8-71.4) | 18.5(1.2-87.9) | 25.2(6.1-70.6) | 0.922 |
| Ventricular pacing (%) | 1.9 (0.2-98.3) | 14.4(0.2-75.1) | 1.6(0.2-98.4) | 0.522 |
| CHA_2_DS_2_-VASc score^i^ | 3 (2-4) | 4 (3-5) | 3 (2-4) | 0.005 |
| HAS-BLED score^j^ | 2 (1-3) | 3 (2-3) | 2 (1-3) | 0.002 |
| C_2_HEST score^k^ | 3 (1-3) | 3 (1-4) | 3 (1-3) | 0.114 |
| HATCH score^l^ | 2 (1-3) | 4 (3-5) | 2 (1-3) | <0.001 |
| Hypertension | 253(80.6%) | 17(94.4%) | 236(79.7%) | 0.215 |
| Diabetes mellitus | 142(45.2%) | 14(77.8%) | 128(43.2%) | 0.006 |
| Hyperlipidemia | 241(76.8%) | 18(100.0%) | 223(75.3%) | 0.010 |
| Chronic obstructive pulmonary disease | 14 (4.5%) | 1(5.6%) | 13(4.4%) | 0.570 |
| Prior stroke | 19(6.1%) | 6(33.3%) | 13(4.4%) | <0.001 |
| Prior myocardial infarction | 57(18.2%) | 5(27.8%) | 52(17.6%) | 0.275 |
| Heart failure |  |  |  | 0.462 |
| Preserved LVEF^m^ | 44(14.0%) | 2(11.1%) | 42(14.2%) |  |
| Reduced LVEF^m^ | 68(21.7%) | 6(33.3%) | 62(20.9%) |  |
| None | 202(64.3%) | 10(55.6%) | 192(64.9%) |  |
| Chronic kidney disease | 108(34.4%) | 9(50.0%) | 99(33.4%) | 0.151 |
| Chronic liver disease | 15(4.8%) | 1(5.6%) | 14(4.7%) | 0.596 |
| Echo parameters |  |  |  |  |
| LVEF^m^ (%) | 66 (53.8-73.0) | 60.0(44.3-72.0) | 66.0(54.0-73.0) | 0.242 |
| Mitral E/e’ | 11.0 (8.0-13.6) | 11.0(9.7-14.3) | 11.0(8.0-13.4) | 0.614 |
| LA^n^ diameter (cm) | 3.8 (3.2-4.1) | 3.8(3.5-4.4) | 3.8(3.2-4.1) | 0.529 |
| RV^o^ systolic function (s’, m/s) | 12.0 (11.0-13.6) | 12.0(10.8-14.0) | 12.0(11.0-13.5) | 0.849 |
| Drug prescribed at baseline |  |  |  |  |
| Antiplatelets | 121(38.5%) | 12(66.7%) | 109(36.8%) | 0.012 |
| Anticoagulants | 30(9.6%) | 1(5.6%) | 29(9.8%) | 1.000 |
| Beta blockers | 122(38.9%) | 6(33.3%) | 116(39.2%) | 0.621 |
| Ivabradine | 25(8.0%) | 3(16.7%) | 22(7.4%) | 0.164 |
| Amiodarone | 58(18.5%) | 2(11.1%) | 56(18.9%) | 0.543 |
| Dronedarone | 4(1.3%) | 2(11.1%) | 2(0.7%) | 0.017 |
| Flecainide | 1(0.3%) | 0(0.0%) | 1(0.3%) | 1.000 |
| Propafenone | 13(4.1%) | 0(0.0%) | 13(4.4%) | 1.000 |
| Digoxin | 5(1.6%) | 0(0.0%) | 5(1.7%) | 1.000 |
| non-DHP CCBs^p^ | 12(3.8%) | 0(0.0%) | 12(4.1%) | 1.000 |
| RAAS^q^ inhibitors | 141(45.0%) | 7(38.9%) | 134(45.4%) | 0.589 |
| Diuretics | 47(15.0%) | 4(22.2%) | 43(14.5%) | 0.325 |
| Statins | 121(38.5%) | 6(33.3%) | 115(38.9%) | 0.640 |
| Metformin | 50(15.9%) | 3(16.7%) | 47(15.9%) | 1.000 |
| SGLT2^r^ inhibitors | 13(4.1%) | 0(0.0%) | 13(4.4%) | 1.000 |
| Follow-up duration (months) | 32 (16-52) | 23.5(11.8-44.5) | 34.0(16.0-52.0) | 0.077 |
| AHRE^s^ Duration**≥**3mins | 125(39.8%) | 15(83.3%) | 110(37.2%) | <0.001 |
| AHRE^s^ Duration**≥**6mins | 103(32.8%) | 12(66.7%) | 91(30.7%) | 0.002 |
| AHRE^s^ Duration**≥**6hrs | 55(17.5%) | 6(33.3%) | 49(16.6%) | 0.069 |
| AHRE^s^ Duration**≥**24hrs | 35(11.1%) | 4(22.2%) | 31(10.5%) | 0.127 |

| Data are presented as medians (interquartile interval) or n (%). Non-parametric continuous variables, as assessed using the Kolmogorov–Smirnov method, were analyzed using the Mann–Whitney U test. Statistical significance is set at p < 0.05.  ^a^MACCE, major cardio/cerebrovascular events  ^b^BMI, body mass index  ^c^PM, pacemaker  ^d^ICD, implantable cardioverter defibrillator  ^e^CRTP, cardiac resynchronization therapy pacemaker  ^f^CRTD, cardiac resynchronization therapy defibrillator  ^g^VT, ventricular tachycardia  ^h^VF, ventricular fibrillation  ^i^ CHA_2_DS_2_-Vasc score: Range from 0 to 9. History of heart failure, hypertension, diabetes, vascular disease, age 65–74 years, and female sex each is calculated as 1 point; 75 years or older and prior stroke, TIA, or thromboembolism each is calculated as 2 points.  ^j^HASBLED score: Range from 0 to 9. Point score is calculated as 1 point each for hypertension, abnormal kidney function, abnormal liver function, prior stroke, prior bleeding or bleeding predisposition, labile international normalized ratio (INR), older than 65 years, medication usage predisposing to bleeding, and alcohol use.  ^k^C_2_HEST score: Range from 0 to 8. C_2_: CAD/COPD (1 point each); H: hypertension (1 point); E: elderly (age ≥ 75 years, 2 points); S: systolic HF (2 points); and T: thyroid disease (hyperthyroidism, 1 point).  ^l^ HATCH score: Range from 0 to 7. Hypertension, 1 point; age >75 years, 1 point; stroke or transient ischemic attack, 2 points; chronic obstructive pulmonary disease, 1 point; heart failure, 2 points.  ^m^LVEF, left ventricular ejection fraction  ^n^LA, left atrium  ^o^RV, right ventricle  ^p^non-DHP CCBs, non-dihydropyridine calcium channel blockers  ^q^RAAS, renin-angiotensin-aldosterone system  ^r^SGLT2, sodium glucose co-transporters 2  ^s^AHRE, atrial high-rate episodes |
| --- |
