# Supplementary material for: HATCH Score and Left Atrial Size Predict Atrial High-Rate Episodes in Patients With Cardiac Implantable Electronic Devices
Source: Front Cardiovasc Med. 2021 Oct 6;8:746225. doi: 10.3389/fcvm.2021.746225 (PMC8528173; doi:10.3389/fcvm.2021.746225)
Supplement: Supplementary file 2 [file Table_2.docx]

**Supplemental Table 2. Multivariate Cox regression analysis for ischemic cerebrovascular events**

| Variables | Model A-1 | | | Model A-2 | | |
| --- | --- | --- | --- | --- | --- | --- |
|  | **HR** | **95%CI** | ***p*** | **HR** | **95%CI** | ***p*** |
| Diabetes mellitus (yes) | 3.273 | 1.071-10.004 | 0.038 | 3.269 | 1.065-10.034 | 0.038 |
| Hyperlipidemia (yes) | 5.793 | 0.000-2.179 | 0.957 | 4.231 | 0.000-1.179 | 0.957 |
| Prior stroke (yes) | 8.421 | 3.007-23.582 | <0.001 | 9.182 | 3.308-25.485 | <0.001 |
| AHRE Duration**≥**3mins | 6.406 | 1.846-22.229 | 0.003 |  |  |  |
| AHRE Duration**≥**6mins |  |  |  | 3.897 | 1.452-10.462 | 0.007 |

| Variables | Model B-1 | | | Model B-2 | | | Model B-3 | | |
| --- | --- | --- | --- | --- | --- | --- | --- | --- | --- |
|  | **HR** | **95%CI** | ***p*** | **HR** | **95%CI** | ***p*** | **HR** | **95%CI** | ***p*** |
| CHA_2_DS_2_-VASc score | 1.651 | 1.196-2.278 | 0.002 |  |  |  |  |  |  |
| HAS-BLED score |  |  |  | 1.769 | 1.145-2.732 | 0.010 |  |  |  |
| HATCH score |  |  |  |  |  |  | 2.870 | 2.064-3.992 | <0.001 |
| AHRE Duration**≥**3mins | 6.307 | 1.819-21.869 | 0.004 | 6.206 | 1.792-21.496 | 0.004 | 2.547 | 0.664-9.769 | 0.173 |
